# Editorial: Mobility, power and the (re)production of inequality and injustice

**DOI:** 10.3389/fsoc.2025.1577768

**Published:** 2025-03-20

**Authors:** Minoo Alinia, Claudia Tazreiter, Vanessa Barker

**Affiliations:** ^1^Department of Sociology, Faculty of Social Sciences, Uppsala University, Uppsala, Sweden; ^2^Division of Migration, Ethnicity and Society, Department of Culture and Society, Liköping University, Linköping, Sweden; ^3^Department of Sociology, Faculty of Social Sciences, Stockholm University, Stockholm, Sweden

**Keywords:** mobility, migration, inequality, power, borders

Mobility is a site of power and stratification. Mobility shapes life chances across and within societies in ways that sociologists are just beginning to identify and explain, particularly as they intersect with class, gender, race, sexuality, and age. Access to mobility through borders, citizenship and nationality rather than receding during globalization has become even more important. What once seemed possible—the rise of a borderless world and the broad recognition of rights–have now regressed under the bitter realities of turbo-nationalism, populism and violent forms of re-bordering. This historical backlash requires new and creative responses to mobility inequalities and injustices. At the same time—and the focus of this Research Topic—it is human mobility across national borders which challenges and unsettles the taken-for-granted patterns of belonging manifest as rights, citizenship, territories, and foundational ideas about justice and about who and what constitutes a society. Mobility and migration across national borders has increased in a way that human kind has never experienced before and now includes very different categories, from labor migration and refugees to elite forms of human mobility. There are different kinds of mobilities and different kinds of mobile subjects. There are those who can decide to leave or to stay and those, who are in fact the majority, who do not have the privilege to do so. And yet, they still try to cross borders and in doing so, challenge the structures, hierarchies, and privileges that block their mobility. Many die trying. Hence, questions such as what categories of mobile people we are talking about, or as Avtar Brah puts it, who is migrating, why, from where, and under what circumstances, are of crucial importance.

Departing from this end, this Research Topic introduces the right to transnational mobility as a site of power and stratification which operates at structural, institutional and individual levels. The Research Topic touches upon some aspects of the current mobility patterns and their relation to the local and global inequalities and associated ideas and assessments of justice and injustice.

Based on their original research the contributing authors problematize transnational mobility in the intersection with other sites of inequalities such as ethnicity, migration, citizenship, class and gender and the way it affects groups and individuals. The problem of mobility expressed in the right or lack of right to cross national borders is discussed both as a symptom of global structural injustices but also as a tool for selection, exclusion and control to sustain and normalize the current order. Contributors investigate new sites of mobility control, ranging from technological innovations, to family reunifications schemes, to conceptions of climate crisis and how it shapes responses to migration.

For example, Weber and Gerald provide one of the first academic studies of Australia's visa cancelation scheme, which relies on new kinds of automated technology to revoke legal permits from legally represent noncitizens to speed up removals from the country. This is a significant shift in migration control as the plan actively seeks to change a person's legal status in order to remove them from the country. But rather than some kind of automated dystopia of efficiency, Weber and Gerland find that a mix of human discretion, human error, and lack of technology create the conditions for illegality and prolong uncertainty, increase detention, deportation, and raise normative questions about fairness and justice.

In this Research Topic two articles focus on aspects of the Swedish state in relation to borders and bordering practices. The core challenges that mobility presents to states in terms of just and rights oriented responses are exemplified by Lundberg in providing an auto ethnographic account of the Swedish refugee rights movement that emerged in the form of the Asylum Commission from 2019 to 2022. This civil society based commission gathered together researchers and activists in a refugee rights movement that also included those with lived experience of the asylum process in Sweden. The work of the commission was in response to more restrictive legislation and administrative approaches to refugees following the “long summer of migration in 2015”. Aligning with global justice oriented authors, Lungberg offers an important assessment of sociolegal alliance building in the face of repressive politics and the ongoing struggles for different futures.

In what may seem like an unlikely source of migration control, Gustafsson and Engblom closely examine changes and growing restrictions to Swedish family reunification. Through their combined set of qualitative interviews with migrants with varying legal statuses, they find family reunification is limited by connections to housing and labor market, contributing to a hierarchies of worthiness among noncitizens rather than rights and recognition.

Focusing on climate-change inducted human migration and displacement, Sim-Sarka argues that the dominant state-centric approaches to migration are wrong-headed, reproducing misunderstandings of the impetus for migration and resulting in long-term inequalities. In her assessment of the interwoven histories leading to this state-of-play, Sim-Sarka critical sites of knowledge production that reproduce the power regime of the state, namely the state as well as researcher and research institutions. Suggesting the need for a new way forward, the article argues for de-exceptionalizing climate-related migration alongside decolonial critiques of the state-of-play. Ultimately the article suggests that a climate-mobilities paradigm is a useful global perspective that can contribute to a reassessment of the protracted inequalities and climate injustice evident in present global geopolitics.

Daneshmehr et al. examine Kolberi (a Kurdish term meaning “carrying on the back”) and its impact on families, particularly mothers. Kolberi refers to the “illegal” cross-border transport of goods, which has become a source of income in the border regions of Iranian and Iraqi Kurdistan. It is highly dangerous and involves numerous physical and emotional risks, including injury, long-term suffering, and even death. Using Pierre Bourdieu's social suffering theory and Robert Park's concept of the marginal man, the study employs qualitative phenomenology to explore the lived experiences of 22 mothers. The findings highlight themes such as physical complications, poverty, marginalization, and dehumanization.

In sum, the articles in this Research Topic contribute to the important and ongoing task of documenting empirically and theorizing the myriad ways in which mobility and borders intersect materially and metaphorically. While myriad examples and experiences across the globe point to increasing securitization of borders and punitive policies and practices toward mobile populations, counter-narratives of hope and justice and practices of solidarity and dissent evidence the possibilities of alternative futures.

